# Epidemiological surveillance of HIV-1 transmitted drug resistance among newly diagnosed individuals in Shijiazhuang, northern China, 2014–2015

**DOI:** 10.1371/journal.pone.0198005

**Published:** 2018-06-05

**Authors:** Xianfeng Wang, Xiaosong Liu, Feng Li, Hong Zhou, Jiefang Li, Yingying Wang, Lihua Liu, Shujun Liu, Yi Feng, Ning Wang

**Affiliations:** 1 Shijiazhuang Center for Disease Control and Prevention, Hebei, P.R. China; 2 National Center for AIDS/STD Control and Prevention, Chinese Center for Disease Control and Prevention, Beijing, P.R. China; University of Cincinnati College of Medicine, UNITED STATES

## Abstract

**Background:**

The widespread use of antiretroviral therapy (ART) has led to considerable concerns about the prevalence of transmitted drug resistance (TDR). Sexual contact, particularly men who have sex with men (MSM) was the most prevalent form of HIV transmission in Shijiazhuang. Hence, we conducted an epidemiological surveillance study on TDR among newly diagnosed individuals who infected-HIV through sexual contact in from 2014–2015.

**Methods:**

Genotypic resistance mutations were defined using the WHO-2009 surveillance list. Potential impact on antiretroviral drug was predicted according to the Stanford HIV db program version 7.0. The role of transmission clusters in drug resistant strains was evaluated by phylogenetic and network analyses.

**Results:**

In this study, 589 individuals were recruited and 542 samples were amplified and sequenced successfully. The over prevalence of TDR was 6.1%: 1.8% to nucleoside reverse transcriptase inhibitors (NRTIs), 2.0% to non- NRTIs (NNRTIs) and 2.4% to protease inhibitors (PIs), respectively. We did not find significant differences in the TDR prevalence by demographic and clinical characteristics (p > 0.05). Using network and phylogenetic analysis, almost 60.0% sequences were clustered together. Of these clusters, 2 included at least two individuals carrying the same resistance mutation, accounting for 21.2% (7/33) individuals with TDR. No significant difference was observed in the clustering rate between the individuals with and without TDR.

**Conclusions:**

We obtained a moderate level TDR rate in studied region. These findings enhance our understanding of HIV-1 drug resistance prevalence in Shijiazhuang, and may be helpful for the comprehensive prevention and control of HIV-1.

## Introduction

The widespread use and increased coverage of ART has reduced significantly the risk of HIV transmission and decreased HIV-related morbidity and mortality[1]. Meanwhile, a major drawback of the global increase in ART access is a corresponding increase in HIV drug resistance, which can be transmitted to newly infected individuals. TDR of HIV has gradually become a particular concern because it has the potential to compromise the efficacy of combination ART regimens and may lead to the failure in first-line ART [2–4].

Previous published studies on TDR investigated in different countries showed that the rates of TDR differs between regions and time period, and the overall rate varies ranging from 0% to 23% in treatment-naïve individuals[5–9]. Although the prevalence of TDR among treatment-naïve HIV-1-infected individuals remains low in most areas of China, some recent reports indicated moderate levels in specific regions[10–12].

Our study focuses on Shijiazhuang city, which is the capital of Hebei province, located in north China, adjacent to Beijing and Tianjin, and Henan province. The first case of HIV-1 infection in Shijiazhuang was diagnosed in 1989, and 2347 HIV/AIDS cases had been cumulatively reported in this region by the end of 2015. The prevalence of HIV infection in the general population in Shijiazhuang city remains at a relatively low level [13]. Data from the sentinel surveillance showed that HIV epidemic among the general population was 0.04% in 2015, but it is spreading rapidly in one or more specific groups in recent years. Currently, sexual contact is the most prevalent form of HIV transmission in China. According to the data of the case registry system, the proportion of sexual transmission has been increasing in the newly reported HIV/AIDS cases, rising from 52.3% in 2005 to 96.7% in 2015 in Shijiazhuang. Furthermore, the trend was particularly apparent for it in men who have sex with men (MSM), which increased from 14.3% in 2005 to 65.7% in 2015.

In China, ART is provided to patients for free through the “Four Free One Care” policy since 2004[14]. Hebei province was also among the first group of free ART provinces in that year. In 2015, the new guideline for ART has been changed to recommend all HIV-diagnosed individuals to receive ART[15]. Up to now, free ART has been available in Shijiazhuang for 13 years. However, there is little data on TDR and other molecular epidemiological data about the HIV epidemic among newly diagnosed, sexually infected individuals in this region. Therefore, we performed an epidemiological surveillance study on TDR among individuals newly diagnosed of HIV-1 infected through sexual contact in Shijiazhuang and aimed to provide these baseline resistance data to guide the choices of initial regimen so as to better clinical management and broader disease control effort.

## Materials and methods

### Ethics statement

All participants provided their written informed consent to participate in this study. Ethical approval was obtained from the local Ethics Committee at Shijiazhuang Center for Disease Control and Prevention in Shijiazhuang city, Hebei province, China.

### Study population

Participants were enrolled if they were diagnosed as HIV seropositive for the first time and infected through sexual contact between January 2014 and December 2015. On obtaining a participant’s informed consent, peripheral blood sample was drawn in an EDTA-added vacutainer tube. CD4^+^ T cell counts were detected using whole blood and plasma samples were aliquoted and stored at -80°C until use. Demographic and behavioral information of participant was collected via counselor’s face-to-face interview.

### CD4^+^ T cells count and HIV-1 viral loads detection

Blood samples were collected within 3 months after infection had been confirmed. According to the manufacturer’s instructions, absolute CD4^+^ T cell count was performed by flow cytometry (FC500, Beckman, USA) within 24 h after blood sample processing using the TRITEST three-color CD4/CD8/CD3 reagent and TRUCOUNT tubes (Becton Dickinson, San Jose, CA). Plasma HIV-1 RNA viral load was determined with NucliSens kit (Biomerieux, France) according to the manufacture’s recommendation.

### Amplification and sequencing of HIV-1 *pol* gene fragment

Viral RNA was extracted from 200 μl of plasma by Silica gel adsorption method (NucliSens® EasyMag®, Biomerieux, France), according to the manufacturer’s instructions. The *pol* gene fragments, containing the entire protease gene (codons 1–99) and partial reverse transcriptase gene (codons 1–255), were amplified by nested PCR as described by Liao et al[4, 16] using primers F1a (5’-TGAARGAITGYACTGARAGRCAGGCTAAT-3’, HXB2:2057–2085) and RT-R1 (5’-ATCCCTGCATAAATCTGACTTGC-3’, HXB2: 3370–3348) for the first round PCR reaction, and PRT-F2 (5’-CTTTARCTTCCCTCARATCACTCT-3’, HXB2: 2243–2266) and RT-R2 (5’- CTTCTGTATGTCATTGACAGTCC-3’, HXB2: 3326–3304) for the second. The PCR products were purified by using a QIAquick Gel Extraction Kit, and then directly sequenced with ABI Prism BigDye Terminator Cycle Sequencing Ready Reaction Kit and ABI 3730 XL sequencer (Applied Biosystems, Foster City, CA). Sequences obtained were assembled and edited by the analysis software, Sequencher 4.8 (Gene Codes Corporation, USA), aligned by using BioEdit v.7.2.

### Drug resistance mutations analysis

All sequences were screened utilizing Calibrated Population Resistance (CPR) tool version 6.0 available at Stanford University HIV Drug Resistance Database (http://cpr.standford.edu/cpr/index.html), and then resistance mutations to NRTIs, NNRTIs, and PIs were defined as the detection of at least one mutation in any drug class according to the WHO surveillance drug resistance mutations (SDRMs) list updated in 2009[17, 18]. The overall prevalence was defined as the percentage of patients harboring any mutations indicative TDR. To predict the susceptibility to available NRTIs, NNRTIs, and PIs, sequences were analyzed using the Stanford HIV db algorithm version 7.0[19]. Three levels of resistance were scored: high (R), intermediate (I, intermediate and low), sensitive (S, potentially resistant and sensitive).

### HIV-1 subtyping

The HIV-1 subtypes and circulating recombinant forms (CRFs) were identified by phylogenetic tree analysis of the *pol* sequences. The edited sequences were aligned with HIV-1 reference sequences available in Los Alamos database (http://www.hiv.lanl.gov). Phylogenetic trees were constructed using the neighbor-joining method based on Kimura two-parameter model with 1000 bootstrap replicates, using MEGA (Molecular Evolutionary Genetic Analysis Software, Version 6.0).

### Phylogenetic analyses and network construction

To avoid the effect of homoplasy of drug resistance mutations on the phylogenetic analysis, all 43 codons associated with major DRM in PR and RT were removed from all of the sequences within the alignment. phylogenetic sequences analysis was estimated via a maximum likelihood (ML) approach with a bootstrap analyses with 1000 replicates using the general time reversible + Gamma (GTR + γ) model of nucleotide substitution in FastTree version 2.1. Transmission cluster were defined as those comprising virus strains from 2 or more individuals whose sequences clustered in phylogenetic trees with a bootstrap value ≥ 70%. The trees were edited and visualized using FigTree version 1.4.2.

To construct the transmission network, sequences were codon-aligned to an HXB2 reference sequence (positions 2253–3749). Forty-eight codons associated with drug resistance were removed from the alignment. Tamura-Nei 93 (TN93) pairwise genetic distance was calculated for all pairs of sequences. The TN93 genetic distance between two sequences ≤1.5% was identified as the potential transmission partners. For visualizing and analyzing network, the network data were processed using software Cytoscape 3.5.2. We described characteristics of the network, including the number of sequences (nodes), links (edges) and clusters (groups of linked sequences) [20–23].

### Statistical analysis

Categorical variables were reported in both numbers and proportions, while continuous variables were calculated as medians ± standard deviation (SD), respectively. χ^2^ tests and logistic regression were used for categorical variables. A two-tailed p values less than 0.05 was considered significant. All data were analyzed using Statistical Analysis System version 9.1 (SAS Institute Inc., USA).

## Results

### Characteristics of the study population

All of 589 patients in the study were newly diagnosed in the year of 2014–2015 and infected through sexual contact. Of all subjects, the majority were male (92.9%, 547/589) and MSM (77.6%, 457/589). The patients studied were of ages ranging from 16 to 78 years with age of 36.5±13.7 years old. 96.9% individuals in this study were Han ethnicity and 82.7% were permanent residents in Shijiazhuang. At enrollment, the mean baseline of CD4^+^ T cell counts was 328±202.3 cells/μL, and the mean of HIV viral RNA load was 4.33±0.9 log copies/mL. The proportional distribution of 589 patients analyzed by sex, age, transmission routes, permanent residence from sociodemographic data were presented in Table 1.

**Table 1:** Cases demographics and HIV-1 subtype distribute (n = 589).

Characteristic	N	%
**Total**	589	100
**Risk behavior**		
MSM	457	77.59
Heterosexual	132	22.41
**Sex**		
Male	547	92.87
Female	42	7.13
**permanent residence**		
Shijiazhuang city	487	82.68
Other	102	17.32
**Age at diagnosis (years)**		
Mean (±SD)	36.5 (±13.7)	
≤45	428	72.67
>45	161	27.33
**Nationality**		
Han	571	96.94
Other	16	2.72
**CD4 cell count (cells/mm3)**		
≤500	483	82.00
>500	106	18.00
Mean CD4 (±SD)	328 (±202.29)	
**HIV-1 RNA Viral load**		
≤1000	46	7.81
>1000	527	89.47
missing	16	2.72
Mean Viral load (log10copies/mL) (±SD)	4.33(±0.94)	
**HIV-1 subtype**		
CRF01_AE	267	49.26
CRF07_BC	151	27.86
B	80	14.76
Other	44	8.12
Unable to amplify	47	

### Distribution of HIV-1 subtypes

Based on RT-PCR and sequencing results, 542 (542/589) samples were amplified and sequenced successfully and the results of subtype and genetic resistance were obtained. Among the study subjects, CRF01_AE was found to be the most common genotype (49.5%, 268/542), followed by CRF07_BC (27.5%, 149/542) and subtype B (14.0%, 76/542, Table 1). These three main HIV-1 subtypes accounted for 91% of infections in this risk group. Other subtypes/CRFs included URF (unique recombinant forms, 5.7%, 31/542), CRF08_BC (0.9%, 5/542), CRF55_01B (0.9%, 5/542) and CRF02_AG (0.7%, 4/542). In addition to these, three CRF65_cpx and one CRF59_01B were also found.

The most prevalent genotypes among heterosexuals were CRF01_AE (37.3%, 44/118), CRF07_BC (27.1%, 32/118), and subtype B (18.6%, 22/118), respectively. While MSM were predominantly infected by CRF01_AE, with 52.8% (224/424) which exceeded than CRF07_BC (27.6%, 117/424) and subtype B (12.7%, 54/424). The distribution of HIV-1 subtypes was significant difference between heterosexuals and MSM (p<0.001).

### Prevalence of transmitted drug resistance (TDR)

**Fig 1:**
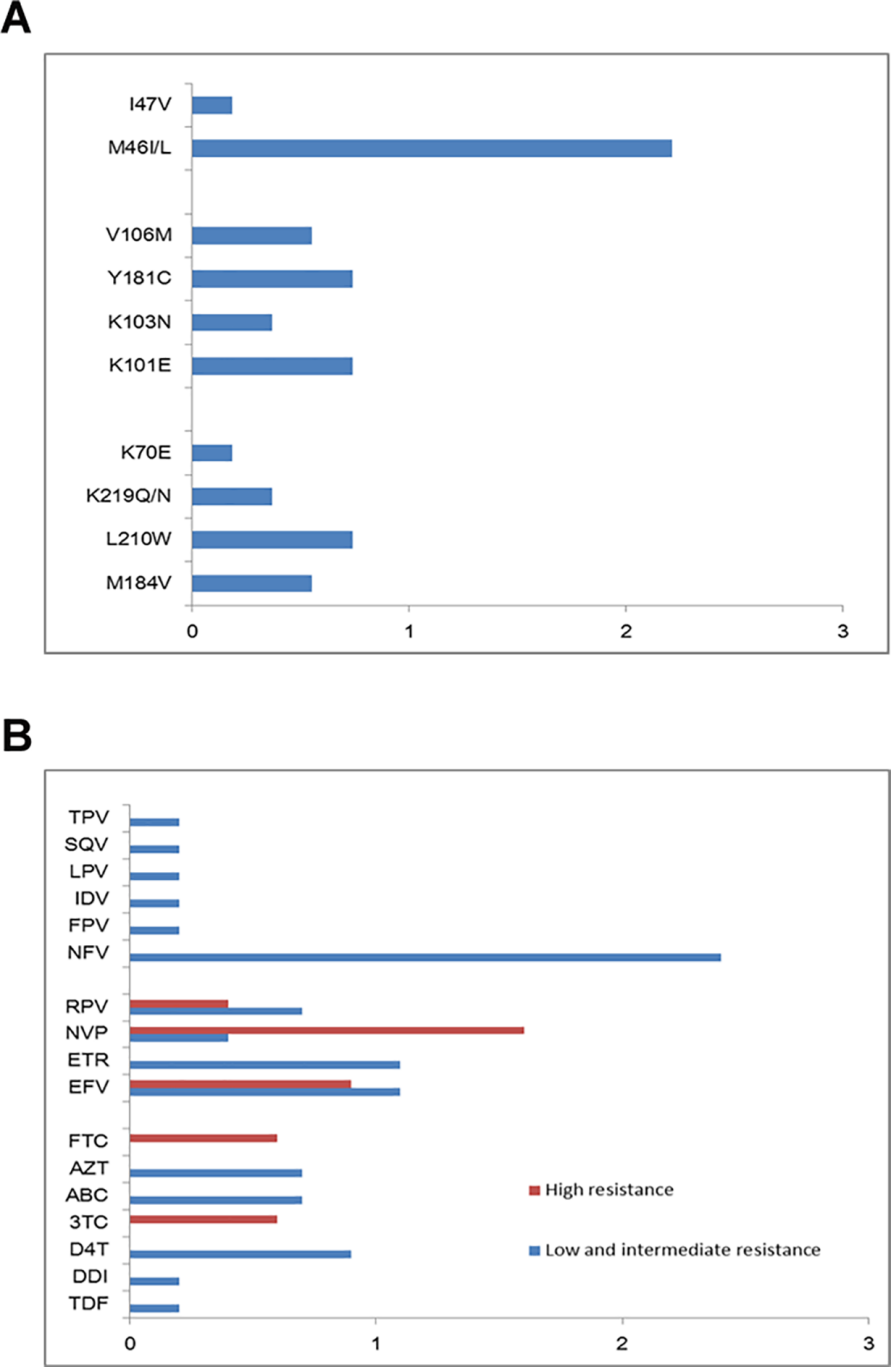
The mutations by drug class and the susceptibility by resistance level. Prevalence of transmitted drug resistance mutations by drug class (A) and predicted susceptibility to antiretroviral drugs by level of resistance (B) among newly diagnosed, infected HIV-1 through sexual contact individuals in Shijiazhuang, 2014–15 (n = 542).

The overall prevalence of TDR in 2014–2015 was 6.1% (33/542; 95% confidence interval (CI): 4.1–8.1%). NRTIs, NNRTIs and PIs were identified among 1.8% (10/542; 95% CI: 0.7–3.0%), 2.0% (11/542; 95% CI: 0.8–3.2%) and 2.4% (13/542; 95% CI: 1.1–3.7%) of the patients, respectively. The majority of subjects with TDR displayed a single drug class resistance mutation (32 out of 33 TDR patients). Only one individual harbored dual classes TDR (M184V and V106M), which conferred resistance to both NRTIs and NNRTIs. No sample with triple classes TDR mutations was found in this study (Table 2). Moreover, three patients carried 2 or more mutations. The M46I/L, PI-associated mutation, was the most frequent mutation observed in 2.2% (12/542) of individuals, while the most commonly observed NNRTIs resistance associated mutations were K101E (0.7%) and Y181C (0.7%), followed by V106M (0.60%), and the most prevalent resistance mutation of NRTIs was L210W(0.70%). An overview of the prevalence of all mutations to NRTIs, NNRTIs and PIs was shown in Fig 1A and S1 Table.

**Table 2:** Comparison of characteristics between newly diagnosed HIV-1-infected patients with and without drug resistance mutations.

	Total	TDR	Wild type	Univariate		Multivariate	
				OR(95% CI)	P-value	OR(95% CI)	P-value
**Patients**	542	33	509				
**Risk behavior**					0.94		0.88
MSM	424	26 (6.1)	398 (93.4)	1		1	
Heterosexual	118	7 (5.9)	111 (94.1)	0.97 (0.41–2.28)		0.92 (0.32–2.67)	
**Sex**					0.92		0.92
Male	507	31 (6.1)	476 (93.9)	1		1	
Female	35	2 (5.7)	33 (94.3)	0.93 (0.21–4.06)		0.92 (0.16–5.24)	
**Age at diagnosis (years)**					0.5		0.95
≤45	389	22 (5.7)	367 (94.3)	1		1	
>45	153	11 (7.2)	142 (92.8)	1.29 (0.61–2.73)		1.03 (0.41–2.59)	
Mean (±SD)	36.8±13.9	38.4±12.2	36.7±14.0		0.51^a^		
**Ethnicity**					0.81		0.72
Han	527	32 (6.1)	495 (93.9)	1		1	
Other	13	1 (7.7)	12 (92.3)	1.29 (0.16–10.23)		1.48 (0.17–12.88)	
**Education**					0.56		0.53
≤9	188	10 (5.3)	178 (94.7)	1		1	
>9	352	23 (6.5)	329 (93.5)	1.26 (0.59–2.70)		1.31 (0.57–3.00)	
**Marriage Status**					0.34		0.48
Single	227	11 (4.8)	216 (95.2)	1		1	
Married	239	14 (5.9)	225 (94.1)	1.22 (0.54–2.75)		1.15 (0.42–3.14)	
Divorced/Widowed	74	8 (10.8)	66 (89.2)	2.38 (0.92–6.16)		2.23 (0.71–6.98)	
**permanent residence**					0.36		0.49
Shijiazhuang city	444	29 (6.5)	414 (93.5)	1		1	
Other	98	4 (4.1)	94 (95.9)	0.61 (0.21–1.77)		0.68 (0.22–2.07)	
**Occupation**					0.28		0.4
students	53	1 (1.9)	52 (98.1)	1		1	
employees	343	20 (5.8)	323 (94.2)	3.22 (0.42–24.51)		2.70 (0.32–22.60)	
unemployed	146	12 (8.2)	134 (91.8)	4.66 (0.59–36.72)		3.74 (0.44–31.62)	
**CD4+ T cell count (cells/mm3)**					0.8		0.68
≤500	452	27 (6.0)	425 (94)	1		1	
>500	90	6 (6.7)	84 (93.3)	0.89 (0.35–2.22)		1.23 (0.46–3.28)	
Mean CD4 (±SD)	320±197.5	309±182.7	321±198.6		0.75^a^		
**Viral load**					0.72		0.81
≤1000	27	2 (7.4)	25 (92.6)	1		1	
>1000	507	30 (5.9)	477 (94.1)	0.79 (0.18–3.48)		0.88 (0.18–4.28)	
Missing	8	1 (12.5)	7 (87.5)	1.79 (0.14–22.7)		1.81 (0.13–24.58)	
Mean log_10_ Viral load (±SD)	4.42±0.88	4.18±1.10	4.43±0.86		0.12^a^		
**Subtype**				1	0.58		0.68
CRF01_AE	267	16 (6.0)	252 (94)	1		1	
CRF07_BC	151	9 (6.0)	140 (94)	0.99 (0.43–2.31)		0.97 (0.40–2.31)	
B	80	7 (9.2)	69 (90.8)	1.50 (0.60–3.80)		1.39 (0.53–3.61)	
Others*	44	1 (2.3)	43 (97.7)	0.36(0.05–2.82)		0.39 (0.05–3.14)	

Data are presented as number (%) of patients, unless otherwise indicated. OR, odds ratio; CI, confidence interval; SD, standard deviation; TDR, transmitted drug resistance; MSM, men who have sex with men.

*Others: including CRF08_BC, CRF02_AG, CRF55_01B, CRF59_01B, CRF65_cpx, CRF_AE/B and URF.

a = two sample t test

### Potential impact of TDR on first-line regimen

The NRTI-associated mutations were predicted to be high-level resistant to Lamivudine (3TC, 0.6%) and Emtricitabine (FTC, 0.6%); intermediate- or low-level resistance to Stavudine (D4T, 0.9%), Azidothymidine (AZT, 0.7%), Abacavir (ABC, 0.7%), Tenofovir (TDF, 0.2%) and Didanosine (DDI, 0.2%). Of 11 patients harboring NNRTIs mutations, all of them were forecasted to have intermediate or high-level resistance to Efavirenz (EFV, 2.0%) and Nevirapine (NVP, 2.0%), followed by Ralpivirine (RPV, 1.1%) and Etravirine (ETR, 1.1%). Among PI-related TDRs, M46I/L was predicted to low resistance to Nelfinavir (NFV, 2.2%) and I47V coferring resisitance for FPV/r(I),IDV/r(I),LPV/r(I), TPV/r(I) and SQV/r(I). (Fig 1B).

### Factors associated with TDR

Comparisons between patients infected with HIV-1 strains harboring drug resistant mutations and those without were shown in Table 2. No differences were observed between the prevalence of TDR and sex, age at diagnosis, nationality, marriage status, education level, permanent residence, Risk behavior, and occupation (all p values > 0.05). Both the mean CD4 cell count and the logarithmic mean of viral load in TDR group were slightly lower than those in without TDR group, although the difference was not statistically significant (p values > 0.05). In addition, no significant different prevalence of TDR were observed among CRF_01AE, CRF_07BC, subtype B, and others subtypes (P = 0.68).

### Phylogenetic and drug resistant-associated transmission networks analysis

**Fig 2:**
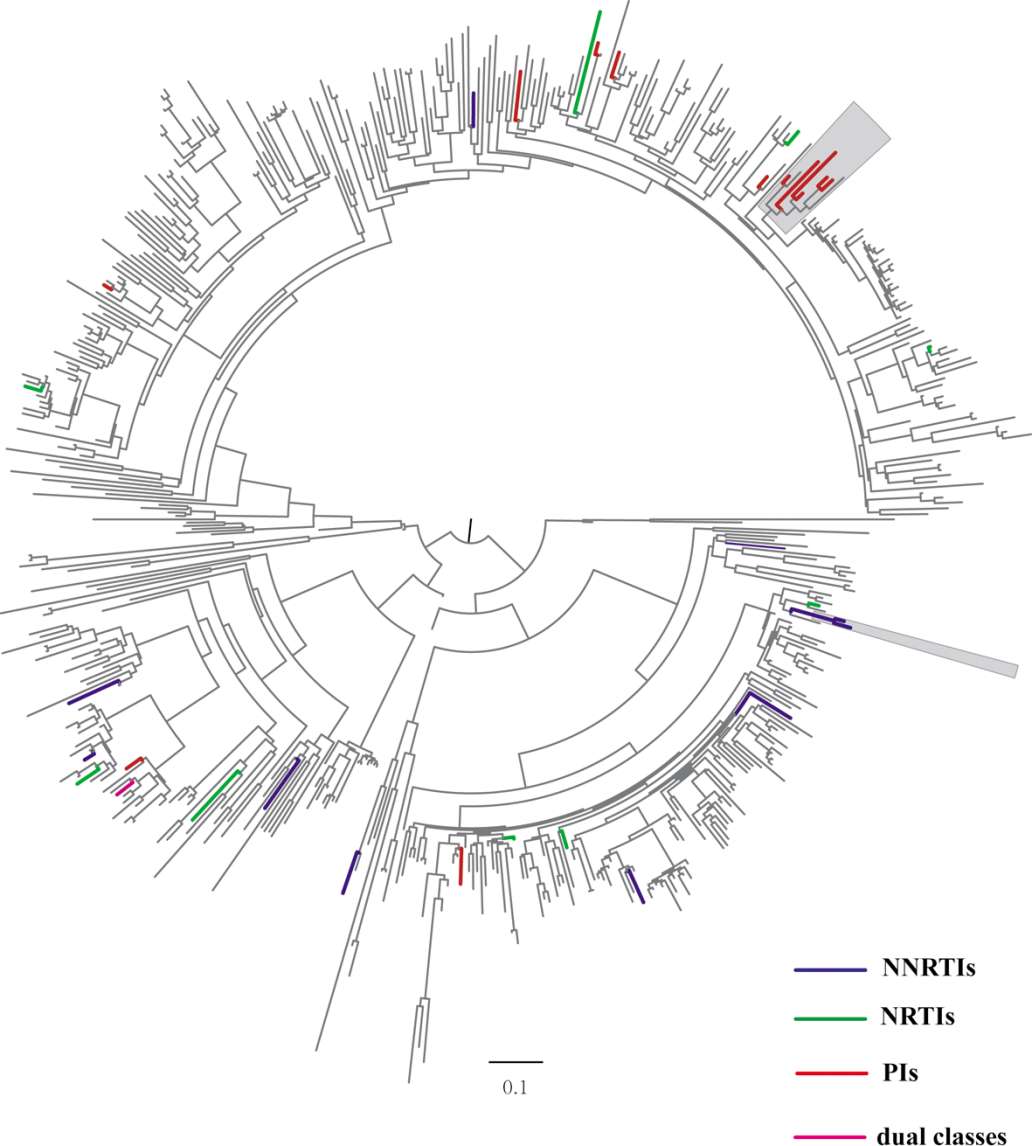
The phylogenetic tree analysis based on HIV-1 *pol* gene sequences. Phylogenetic tree constructed based on HIV-1 partial *pol* fragment (1060bp) obtained from plasma samples of 542 patients who treatment-naïve, sexually HIV-1-infected. Forty-three codons associated with resistance mutations were remove from the alignment. The maximum likelihood phylogenetic tree of all of available sequences was construction using GTR + Gamma nucleotide model in FastTree package. Bootstrap with 1000 replicated was applied to evaluate the reliability of the reconstructed tree, and bootstrap values ≥70 were shown at nodes on the tree. Colored branches represent sequences harboring resistant-drug mutations and grey box indicates the identified clusters which contain at least two individuals sharing the same mutation. Red: PIs; blue: NNRTIs; green: NRTIs; purple: dual classes.

Phylogenetic analysis showed that 325 (60.0%) of 542 sequences of this study grouped in 72 transmission clusters composed of 2 or more individuals. 15 (45.5%) out of 33 sequences with TDR were included in 8 clusters. of which, 2 clusters included at least two individuals carrying the same resistance mutation, the M46I/L was one of the sharing mutation, and K101E was the other (Fig 2). The prevalence of TDR was not significantly different among individuals who were part of clusters and who were not [4.6% (15/325) vs. 8.3% (18/217), respectively; P = 0.1].

**Fig 3:**
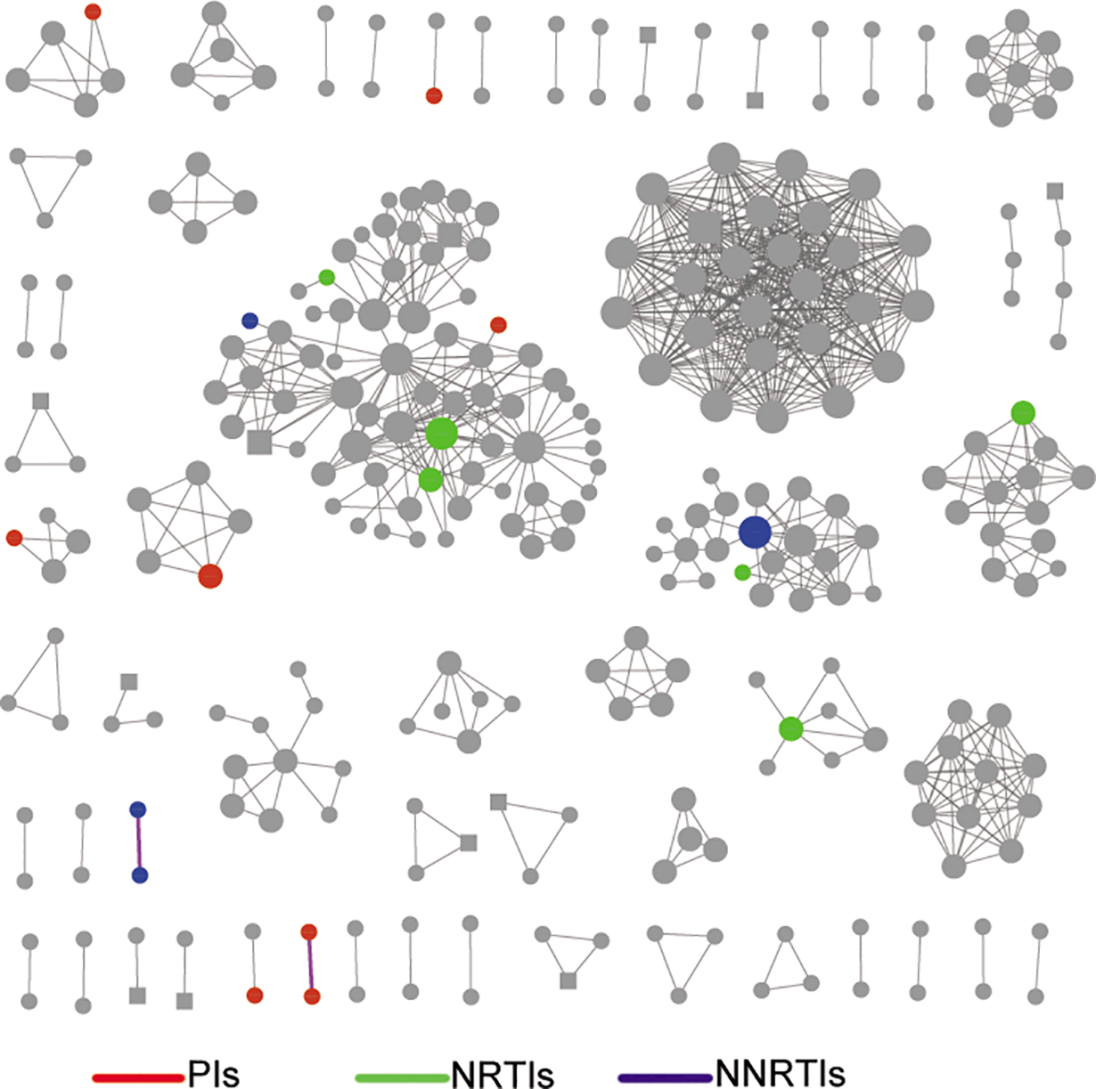
HIV-1 clusters with evidence of transmitted drug resistance (TDR). Individuals shared a same drug resistance mutation (DRM) with a potential transmission partner. Circles represent male and squares are female. Colored nodes represent individual harboring resistant-drug mutations and purple edges indicate a shared DRM. Red: PIs; blue: NNRTIs; green: NRTIs.

To explore the effect of TDR on viral transmission based on the transmission network, we also construct transmission network. Similar to the phylogenetic analysis, 57 clusters were identified, of which 11 contained at least one individual with TDR. Among 542 individuals in the network, 301 (55.5%) were potential transmission partners with another, and 18 (54.5%) individuals harboring mutations of drug-resistance were included (Fig 3). No significant difference was observed in the clustering rate between the individuals with and without TDR (P = 1.0).

## Discussion

In the present study, we analyzed the HIV-1 *pol* gene sequences of 542 patients with newly diagnosed HIV-1-infection through sexual contact and treatment-naïve in Shijiazhuang City from 2014–2015. The studied samples represent 67.4% of 804 identified patients (data from Shijiazhuang CDC) during this period.

Results of phylogenetic analysis illustrated the diversity of HIV-1 subtype among the studied subjects. A total of nine HIV-1 subtypes, including CRF01_AE, CRF07_BC, B, CRF08_BC, CRF02_AG, CRF55_01B, CRF59_01B, and CRF65_cpx, and URFs, were detected. CRF01_AE, CRF07_BC, and subtype B represented the three most common subtypes among sexual contact population, similar to what has been shown in other reports [24–27]. In addition, CRF59_01B and CRF65_cpx were firstly identified in our samples in Shijiazhuang. These finding indicated the highly genetic heterogeneity and subtypes/CRFs diversity among sexual contact population epidemic in this city, which may facilitated the more generation of new recombination forms and made intervention more complicated.

The overall prevalence among 2014–2015 studied samples was 6.1% in Shijiazhuang city, a moderate level (5%-15%) according to the WHO categorization method. The TDR rate was slightly higher than that of studies in other region in China [26, 28, 29], while lower than that previous published studies in some industrialized countries. The higher prevalence of TDR in developed countries may be due to the fact that the patients there have been subject to more prolonged periods of ART in these countries[30, 31]. When examining the presence of resistance by drug class, we found that there was no significant difference in the prevalence of drug-resistant mutations among the three antiretroviral classes (NRTI, 1.8%; NNRTI, 2.0%; and PI, 2.4%). For PIs mutations, 91.7% of M46I/L (11/12) was detected in CRF01_AE-infected MSM in our study. Although the M46L mutation can decrease susceptibility to a number of protease inhibitors when present with other mutations, it is also a natural polymorphism in CRF_01 AE. It is likely that the occurrence of M46I/L may be the characteristics of specific viral, and may not have any particular impact on the success of current ART [32–34]. In NNRTIs class, high-level resistant to Efavirenz and Nevirapine were mainly due to the mutations K103N, V106M and Y181C. For NRTIs, 0.6% of patients were predicted to be resistant to Lamivudine and Emtricitabine, which is mostly attributable to the presence of M184V. Lamivudine, Efavirenz and Nevirapine have been prescribed in the first-line regiment of ART in China [10, 35]. We therefore considered that the emergence of these mutations may relate to the usage of first-line regimen.

No significant differences were observed between the prevalence of TDR and demographic characteristic, which was similar with a previous study [36, 37]. Many studies reported that the higher prevalence of TDR among MSM compared to heterosexuals; interestingly, the opposite results also were found in others reports [38–40]. In this study, the overall prevalence of TDR was not significantly different between MSM and heterosexual. Since the majority of newly diagnosed HIV-1-infected individuals were MSM (78%) in Shijiazhuang, TDR surveillance in this group should be performed to better inform treatment decisions.

This study had a clustering rate with 60% of sequences segregating 72 clusters using phylogenetic analysis. with regard to the transmission of resistant variants in transmission clusters, only 2 transmission clusters were identified that contained drug-resistant individuals having a transmission relationship with each other. The remaining individuals carrying TDR in the network were independent. There was no closely correlation between the clustering rate and the spread of TDR. In previous study [41], the contribution of transmission cluster to the spread has been reported at frequency of 20% in newly diagnosed patients, similar to our study results.

In summary, we focused on the prevalence of TDR among individuals who were newly diagnosed and infected-HIV through sexually contact in Shijiazhuang in 2014–2015. The overall prevalence of TDR was 6.1%, which distributed almost equally among the three drug classes (NRTI, NNRTI and PI) as a moderate level. Although TDR was identified in the transmission clusters, most of TDR were independent rather than converged together. These findings enhance our understanding of HIV-1 drug resistance prevalence and subtype distribution in Shijiazhuang.

## Supporting information

S1 Table The characteristic of individuals with transmitted HIVDR mutations (SDRMs). (DOCX)

## Abbreviations

ART: Antiretroviral therapy
ARV: Antiretroviral
CRF: Circulating recombinant form
HIV: Human immunodeficiency virus
NNRTIs: Non-nucleoside reverse transcriptase inhibitors
NRTIs: Nucleoside reverse transcriptase inhibitors
PIs: Protease inhibitors
TDR: Transmitted drug resistance
URF: Unique recombinant form

## References

[1] Lima VD, Harrigan R, Bangsberg DR, Hogg RS, Gross R, Yip B, et al The Combined Effect of Modern Highly Active Antiretroviral Therapy Regimens and Adherence on Mortality Over Time. Journal of acquired immune deficiency syndromes. 2009;50(5):529–36. doi: 10.1097/QAI.0b013e31819675e9 PubMed PMID: PMC3606956. 19223785 10.1097/QAI.0b013e31819675e9 PMC3606956

[2] The HIVCC. The effect of combined antiretroviral therapy on the overall mortality of HIV-infected individuals. Aids. 2010;24(1):123–37. doi: 10.1097/QAD.0b013e3283324283 PubMed PMID: PMC2920287. 19770621 10.1097/QAD.0b013e3283324283 PMC2920287

[3] Yang J, Xing H, Niu J, Liao L, Ruan Y, He X, et al The emergence of HIV-1 primary drug resistance genotypes among treatment-naïve men who have sex with men in high-prevalence areas in China. Archives of virology. 2013;158:839–44. doi: 10.1007/s00705-012-1557-7 23224760 10.1007/s00705-012-1557-7

[4] Liao L, Xing H, Shang H, Li JY, Zhong P, Cheng H, et al The prevalence of transmitted antiretroviral drug resistance in treatment naïve HIV-infected individuals in China. Journal of acquired immune deficiency syndromes. 2010;53(Suppl 1):S10–S4. doi: 10.1097/qai 20104099 10.1097/QAI.0b013e3181c7d363 PMC3422681

[5] Booth CL, Geretti AM. Prevalence and determinants of transmitted antiretroviral drug resistance in HIV-1 infection. The Journal of antimicrobial chemotherapy. 2007;59(6):1047–56. doi: 10.1093/jac/dkm082 . 17449483 10.1093/jac/dkm082

[6] Todesco E, Charpentier C, Bertine M, Wirden M, Storto A, Desire N, et al Disparities in HIV-1 transmitted drug resistance detected by ultradeep sequencing between men who have sex with men and heterosexual populations. HIV medicine. 2017;18(9):696–700. doi: 10.1111/hiv.12508 . 28444829 10.1111/hiv.12508

[7] Onywera H, Maman D, Inzaule S, Auma E, Were K, Fredrick H, et al Surveillance of HIV-1 pol transmitted drug resistance in acutely and recently infected antiretroviral drug-naive persons in rural western Kenya. PloS one. 2017;12(2):e0171124 doi: 10.1371/journal.pone.0171124 ; PubMed Central PMCID: PMC5298248. 28178281 10.1371/journal.pone.0171124 PMC5298248

[8] Li H, Chang S, Han Y, Zhuang D, Li L, Liu Y, et al The prevalence of drug resistance among treatment-naive HIV-1-infected individuals in China during pre- and post- 2004. BMC infectious diseases. 2016;16(1):605 doi: 10.1186/s12879-016-1928-x ; PubMed Central PMCID: PMC5080753. 27782811 10.1186/s12879-016-1928-x PMC5080753

[9] Lu XL, Kang XJ, Liu YJ, Li Y, Chen SL, Li JY, et al Surveillance of Transmitted Drug Resistance in HIV-1-Infected Youths Aged 16 to 25 Years, a Decade After Scale-up of Antiretroviral Therapy in Hebei, China. AIDS research and human retroviruses. 2017;33(4):359–64. doi: 10.1089/AID.2016.0231 27750023 10.1089/AID.2016.0231

[10] Li L, Sun B, Zeng H, Sun Z, Sun G, Yang R. Relatively high prevalence of drug resistance among antiretroviral-naive patients from Henan, Central China. AIDS research and human retroviruses. 2014;30(2):160–4. doi: 10.1089/AID.2013.0144 ; PubMed Central PMCID: PMC3910477. 23800338 10.1089/aid.2013.0144 PMC3910477

[11] Li L, Sun G, Liang S, Li J, Li T, Wang Z, et al Different distribution of HIV-1 subtype and drug resistance were found among treatment naive individuals in Henan, Guangxi, and Yunnan province of China. PloS one. 2013;8(10):e75777 doi: 10.1371/journal.pone.0075777 ; PubMed Central PMCID: PMC3789720. 24098398 10.1371/journal.pone.0075777 PMC3789720

[12] Zhao B, Han XX, Xu JJ, Hu Q, Chu Z, Zhang J, et al Increase of RT-Related Transmitted Drug Resistance in Non-CRF01_AE Among HIV Type 1–Infected Men Who Have Sex With Men in the 7 Cities of China. Journal of acquired immune deficiency syndromes. 2015;68(3):250–5. doi: 10.1097/QAI.0000000000000467 25469530 10.1097/QAI.0000000000000467

[13] Li L, Lu X, Li H, Chen L, Wang Z, Liu Y, et al High genetic diversity of HIV-1 was found in men who have sex with men in Shijiazhuang, China. Infection, genetics and evolution: journal of molecular epidemiology and evolutionary genetics in infectious diseases. 2011;11(6):1487–92. doi: 10.1016/j.meegid.2011.05.017 21645646 10.1016/j.meegid.2011.05.017

[14] Zhang FJ, Pan J, Yu L, Wen Y, Zhao Y. Current progress of China’s free ART program. Cell Research. 2005;15(11–12):877–82 doi: 10.1038/sj.cr.7290362 16354563 10.1038/sj.cr.7290362

[15] Hao M, Wang J, Xin R, Li X, Hao Y, Chen J, et al Low Rates of Transmitted Drug Resistances Among Treatment-Naive HIV-1-infected Students in Beijing, China. AIDS research and human retroviruses. 2017;33(9):970–6. doi: 10.1089/AID.2017.0053 . 28325065 10.1089/AID.2017.0053

[16] Zhang J, Shen ZY, Li Z, Liang SJ, He C, Liang FX, et al Genetic Characteristics of CRF01_AE Among Newly Diagnosed HIV-1-Infected 16- to 25-Year Olds in 3 Geographic Regions of Guangxi, China. Medicine. 2015;94(21):e894 doi: 10.1097/MD.0000000000000894 ; PubMed Central PMCID: PMC4616409. 26020400 10.1097/MD.0000000000000894 PMC4616409

[17] Bennett DE, Camacho RJ, Otelea D, Kuritzkes DR, Fleury H, Kiuchi M, et al Drug resistance mutations for surveillance of transmitted HIV-1 drug-resistance: 2009 update. PloS one. 2009;4(3):e4724 doi: 10.1371/journal.pone.0004724 ; PubMed Central PMCID: PMC2648874. 19266092 10.1371/journal.pone.0004724 PMC2648874

[18] Gifford RJ, Liu TF, Rhee SY, Kiuchi M, Hue S, Pillay D, et al The calibrated population resistance tool: standardized genotypic estimation of transmitted HIV-1 drug resistance. Bioinformatics. 2009;25(9):1197–8. doi: 10.1093/bioinformatics/btp134 ; PubMed Central PMCID: PMC2672634. 19304876 10.1093/bioinformatics/btp134 PMC2672634

[19] Tang MW, Liu TF, Shafer RW. The HIVdb System for HIV-1 Genotypic Resistance Interpretation. Intervirology. 2012;55(2):98–101. doi: 10.1159/000331998 22286876 10.1159/000331998 PMC7068798

[20] Wertheim JO, Oster AM, Johnson JA, Switzer WM, Saduvala N, Hernandez AL, et al Transmission fitness of drug-resistant HIV revealed in a surveillance system transmission network. Virus evolution. 2017;3(1):vex008 doi: 10.1093/ve/vex008 ; PubMed Central PMCID: PMC5399924. 28458918 10.1093/ve/vex008 PMC5399924

[21] Wertheim JO, Leigh Brown AJ, Hepler NL, Mehta SR, Richman DD, Smith DM, et al The global transmission network of HIV-1. The Journal of infectious diseases. 2014;209(2):304–13. doi: 10.1093/infdis/jit524 ; PubMed Central PMCID: PMC3873788. 24151309 10.1093/infdis/jit524 PMC3873788

[22] Wertheim JO, Kosakovsky Pond SL, Forgione LA, Mehta SR, Murrell B, Shah S, et al Social and Genetic Networks of HIV-1 Transmission in New York City. PLoS pathogens. 2017;13(1):e1006000 doi: 10.1371/journal.ppat.1006000 ; PubMed Central PMCID: PMC5221827 following competing interests: JOW is a paid consultant for the Centers for Disease Control and Prevention. 28068413 10.1371/journal.ppat.1006000 PMC5221827

[23] Oster AM, Wertheim JO, Hernandez AL, Ocfemia MC, Saduvala N, Hall HI. Using Molecular HIV Surveillance Data to Understand Transmission Between Subpopulations in the United States. Journal of acquired immune deficiency syndromes. 2015;70(4):444–51. doi: 10.1097/QAI.0000000000000809 ; PubMed Central PMCID: PMC4878401. 26302431 10.1097/QAI.0000000000000809 PMC4878401

[24] Li X, Xue Y, Cheng H, Lin Y, Zhou L, Ning Z, et al HIV-1 Genetic Diversity and Its Impact on Baseline CD4+T Cells and Viral Loads among Recently Infected Men Who Have Sex with Men in Shanghai, China. PloS one. 2015;10(6):e0129559 doi: 10.1371/journal.pone.0129559 ; PubMed Central PMCID: PMC4486722. 26121491 10.1371/journal.pone.0129559 PMC4486722

[25] Feng Y, He X, Hsi JH, Li F, Li X, Wang Q, et al The rapidly expanding CRF01_AE epidemic in China is driven by multiple lineages of HIV-1 viruses introduced in the 1990s. Aids. 2013;27(11):1793–802. doi: 10.1097/QAD.0b013e328360db2d ; PubMed Central PMCID: PMC3819312. 23807275 10.1097/QAD.0b013e328360db2d PMC3819312

[26] Chen M, Ma Y, Su Y, Yang L, Zhang R, Yang C, et al HIV-1 Genetic Characteristics and Transmitted Drug Resistance among Men Who Have Sex with Men in Kunming, China. PloS one. 2014;9(1):e87033 doi: 10.1371/journal.pone.0087033 24489829 10.1371/journal.pone.0087033 PMC3906090

[27] Zhao J, Chen L, Chaillon A, Zheng C, Cai W, Yang Z, et al The dynamics of the HIV epidemic among men who have sex with men (MSM) from 2005 to 2012 in Shenzhen, China. Scientific reports. 2016;6:28703 doi: 10.1038/srep28703 ; PubMed Central PMCID: PMC4926087. 27352965 10.1038/srep28703 PMC4926087

[28] Dai L, Li N, Wei F, Li J, Liu Y, Xia W, et al Transmitted antiretroviral drug resistance in the men who have sex with men HIV patient cohort, Beijing, China, 2008–2011. Viral immunology. 2014;27(8):392–7. doi: 10.1089/vim.2014.0025 ; PubMed Central PMCID: PMC4183911. 25084305 10.1089/vim.2014.0025 PMC4183911

[29] Liao L, Xing H, Dong Y, Qin G, Ma Y, Lu H, et al Surveys of transmitted HIV drug resistance in 7 geographic Regions in China, 2008–2009. Clinical infectious diseases: an official publication of the Infectious Diseases Society of America. 2012;54 Suppl 4:S320–3. doi: 10.1093/cid/cir1016 . 22544196 10.1093/cid/cir1016

[30] Tostevin A, White E, Dunn D, Croxford S, Delpech V, Williams I, et al Recent trends and patterns in HIV-1 transmitted drug resistance in the United Kingdom. HIV medicine. 2017;18(3):204–13. doi: 10.1111/hiv.12414 ; PubMed Central PMCID: PMC5297994. 27476929 10.1111/hiv.12414 PMC5297994

[31] Hattori J, Shiino T, Gatanaga H, Mori H, Minami R, Uchida K, et al Characteristics of Transmitted Drug-Resistant HIV-1 in Recently Infected Treatment-Naive Patients in Japan. Journal of acquired immune deficiency syndromes. 2016;71(4):367–73 doi: 10.1097/QAI.0000000000000861 26428230 10.1097/QAI.0000000000000861

[32] Hofstra LM, Sauvageot N, Albert J, Alexiev I, Garcia F, Struck D, et al Transmission of HIV Drug Resistance and the Predicted Effect on Current First-line Regimens in Europe. Clinical infectious diseases: an official publication of the Infectious Diseases Society of America. 2016;62(5):655–63. doi: 10.1093/cid/civ963 ; PubMed Central PMCID: PMC4741360. 26620652 10.1093/cid/civ963 PMC4741360

[33] Wang X, Wu Y, Mao L, Xia W, Zhang W, Dai L, et al Targeting HIV Prevention Based on Molecular Epidemiology Among Deeply Sampled Subnetworks of Men Who Have Sex With Men. Clinical infectious diseases: an official publication of the Infectious Diseases Society of America. 2015;61(9):1462–8. doi: 10.1093/cid/civ526 ; PubMed Central PMCID: PMC4599390. 26129754 10.1093/cid/civ526 PMC4599390

[34] Xing H, Ruan Y, Hsi JH, Kan W, Liao L, Leng X, et al Reductions in virological failure and drug resistance in Chinese antiretroviral-treated patients due to lamivudine-based regimens, 2003–12. Journal of Antimicrobial Chemotherapy. 2015;70:2097–103. doi: 10.1093/jac/dkv078 25855758 10.1093/jac/dkv078

[35] Lin B, Sun X, Su S, Lv C, Zhang X, Lin L, et al HIV drug resistance in HIV positive individuals under antiretroviral treatment in Shandong Province, China. PloS one. 2017;12(7):e0181997 doi: 10.1371/journal.pone.0181997 PubMed PMID: PMC5531464. 28750025 10.1371/journal.pone.0181997 PMC5531464

[36] Sallam M, Sahin GO, Indriethason H, Esbjornsson J, Love A, Widell A, et al Decreasing prevalence of transmitted drug resistance among ART-naive HIV-1-infected patients in Iceland, 1996–2012. Infection ecology & epidemiology. 2017;7(1):1328964 doi: 10.1080/20008686.2017.1328964 ; PubMed Central PMCID: PMC5475329. 28649306 10.1080/20008686.2017.1328964 PMC5475329

[37] Hauser A, Hofmann A, Hanke K, Bremer V, Bartmeyer B, Kuecherer C, et al National molecular surveillance of recently acquired HIV infections in Germany, 2013 to 2014. Euro surveillance: bulletin Europeen sur les maladies transmissibles = European communicable disease bulletin. 2017;22(2). doi: 10.2807/1560-7917.ES.2017.22.2.30436 ; PubMed Central PMCID: PMC5404484. 28105988 10.2807/1560-7917.ES.2017.22.2.30436 PMC5404484

[38] Velasco-de-Castro CA, Grinsztejn B, Veloso VG, Bastos FI, Pilotto JH, Fernandes N, et al HIV-1 diversity and drug resistance mutations among people seeking HIV diagnosis in voluntary counseling and testing sites in Rio de Janeiro, Brazil. PloS one. 2014;9(1):e87622 doi: 10.1371/journal.pone.0087622 ; PubMed Central PMCID: PMC3907471. 24498155 10.1371/journal.pone.0087622 PMC3907471

[39] Panichsillapakit T, Smith DM, Wertheim JO, Richman DD, Little SJ, Mehta SR. Prevalence of Transmitted HIV Drug Resistance Among Recently Infected Persons in San Diego, CA 1996–2013. Journal of acquired immune deficiency syndromes. 2016;71(2):228–36. doi: 10.1097/QAI.0000000000000831 ; PubMed Central PMCID: PMC4712087. 26413846 10.1097/QAI.0000000000000831 PMC4712087

[40] Su Y, Zhang F, Liu H, Smith MK, Zhu L, Wu J, et al The prevalence of HIV-1 drug resistance among antiretroviral treatment naive individuals in mainland China: a meta-analysis. PloS one. 2014;9(10):e110652 doi: 10.1371/journal.pone.0110652 ; PubMed Central PMCID: PMC4208788. 25343483 10.1371/journal.pone.0110652 PMC4208788

[41] Vega Y, Delgado E, Fernandez-Garcia A, Cuevas MT, Thomson MM, Montero V, et al Epidemiological Surveillance of HIV-1 Transmitted Drug Resistance in Spain in 2004–2012: Relevance of Transmission Clusters in the Propagation of Resistance Mutations. PloS one. 2015;10(5):e0125699 doi: 10.1371/journal.pone.0125699 ; PubMed Central PMCID: PMC4444345. 26010948 10.1371/journal.pone.0125699 PMC4444345

