# Supplementary material for: Epidemiological surveillance of HIV-1 transmitted drug resistance among newly diagnosed individuals in Shijiazhuang, northern China, 2014–2015
Source: PLoS One. 2018 Jun 5;13(6):e0198005. doi: 10.1371/journal.pone.0198005 (PMC5988301; doi:10.1371/journal.pone.0198005)
Supplement: S1 Table — (DOCX) [file pone.0198005.s001.docx]

Table S1 The characteristic of Individuals with Transmitted HIVDR Mutations (SDRMs)

| **Sanple ID** | **Gender** | **Age** | **Transmission routes** | **Subtype** | **Resistance mutations** | | |
| --- | --- | --- | --- | --- | --- | --- | --- |
|  |  |  |  |  | **PI** | **NRTI** | **NNRTI** |
| 14sjz003 | M | 45 | MSM | CRF01_AE | M46I |  |  |
| 14sjz007 | M | 48 | MSM | CRF01_AE | M46L |  |  |
| 14sjz079 | M | 42 | MSM | CRF01_AE | M46I |  |  |
| 14sjz117 | M | 21 | MSM | CRF01_AE | M46L |  |  |
| 14sjz167 | M | 23 | MSM | CRF01_AE | M46L |  |  |
| 14sjz216 | M | 38 | MSM | CRF01_AE | M46L |  |  |
| 14sjz228 | M | 32 | MSM | CRF01_AE | M46I |  |  |
| 15sjz047 | M | 47 | Heterosexual | B | M46I |  |  |
| 15sjz092 | M | 28 | MSM | CRF01_AE | M46L |  |  |
| 15sjz146 | M | 55 | MSM | CRF01_AE | M46L |  |  |
| 15sjz171 | M | 24 | MSM | CRF01_AE | M46L |  |  |
| 15sjz306 | M | 53 | MSM | CRF01_AE | M46L |  |  |
| 14sjz005 | M | 28 | MSM | CRF07_BC | I47V | |  |
| 14sjz192 | M | 44 | MSM | CRF01_AE |  | L210W |  |
| 14sjz254 | M | 34 | Heterosexual | CRF01_AE |  | L210W |  |
| 15sjz023 | M | 25 | MSM | CRF01_AE |  | L210W |  |
| 15sjz089 | M | 28 | MSM | B |  | L210W |  |
| 14sjz247 | M | 48 | MSM | B |  | K219Q |  |
| 15sjz142 | M | 42 | MSM | CRF07_BC |  | K219N |  |
| 15sjz102 | M | 21 | MSM | CRF07_BC |  | K70E |  |
| 14sjz168 | M | 34 | MSM | CRF07_BC |  | M184V |  |
| 15sjz303 | M | 51 | MSM | CRF01_AE |  | M184V |  |
| 14sjz264 | M | 62 | Heterosexual | B |  | M184V | V106M |
| 14sjz114 | M | 29 | MSM | B |  |  | V106M |
| 15sjz010 | F | 34 | Heterosexual | CRF01_AE |  |  | V106M |
| 14sjz119 | M | 23 | MSM | B |  |  | K103N |
| 14sjz173 | M | 50 | Heterosexual | B |  |  | K103N |
| 14sjz050 | M | 18 | MSM | CRF07_BC |  |  | Y181C |
| 14sjz113 | M | 43 | Heterosexual | CRF07_BC |  |  | Y181C |
| 14sjz224 | M | 46 | MSM | CRF07_BC |  |  | K101E |
| 15sjz094 | F | 42 | Heterosexual | CRF08_BC |  |  | K101E |
| 15sjz212 | M | 62 | MSM | CRF07_BC |  |  | K101E, Y181C |
| 15sjz225 | M | 46 | MSM | CRF07_BC |  |  | K101E, Y181C |

PI: protease inhibitor; NRTI: nucleotide reverse transcriptase inhibitor; NNRTI: non-nucleoside reverse transcriptase inhibitor
